# Solid-state NMR of paired helical filaments formed by the core tau fragment tau(297-391)

**DOI:** 10.3389/fnins.2022.988074

**Published:** 2022-12-08

**Authors:** Youssra K. Al-Hilaly, Connor Hurt, Janet E. Rickard, Charles R. Harrington, John M. D. Storey, Claude M. Wischik, Louise C. Serpell, Ansgar B. Siemer

**Affiliations:** ^1^Sussex Neuroscience, School of Life Sciences, University of Sussex, Falmer, United Kingdom; ^2^Chemistry Department, College of Science, Mustansiriyah University, Baghdad, Iraq; ^3^Department of Physiology and Neuroscience, Zilkha Neurogenetic Institute, Keck School of Medicine, University of Southern California, Los Angeles, CA, United States; ^4^Institute of Medical Sciences, University of Aberdeen, Aberdeen, United Kingdom; ^5^TauRx Therapeutics Ltd., Aberdeen, United Kingdom; ^6^Department of Chemistry, University of Aberdeen, Aberdeen, United Kingdom

**Keywords:** tau protein, solid-state NMR, amyloid structure, neurodegenerative diseases, Alzheimer’s disease

## Abstract

Aggregation of the tau protein into fibrillar cross-β aggregates is a hallmark of Alzheimer’s diseases (AD) and many other neurodegenerative tauopathies. Recently, several core structures of patient-derived tau paired helical filaments (PHFs) have been solved revealing a structural variability that often correlates with a specific tauopathy. To further characterize the dynamics of these fibril cores, to screen for strain-specific small molecules as potential biomarkers and therapeutics, and to develop strain-specific antibodies, recombinant in-vitro models of tau filaments are needed. We recently showed that a 95-residue fragment of tau (from residue 297 to 391), termed dGAE, forms filaments *in vitro* in the absence of polyanionic co-factors often used for *in vitro* aggregation of full-length tau. Tau(297-391) was identified as the proteolytic resistant core of tau PHFs and overlaps with the structures characterized by cryo-electron microscopy in *ex vivo* PHFs, making it a promising model for the study of AD tau filaments *in vitro*. In the present study, we used solid-state NMR to characterize tau(297-391) filaments and show that such filaments assembled under non-reducing conditions are more dynamic and less ordered than those made in the presence of the reducing agent DTT. We further report the resonance assignment of tau(297-391)+DTT filaments and compare it to existing core structures of tau.

## Introduction

Tau accumulates intracellularly in neurofibrillary tangles as paired helical and straight filaments in AD and tau also accumulates as filaments in several other neurodegenerative diseases collectively known as tauopathies (Vaquer-Alicea et al., 2021). The presence of tau filaments is strongly correlated with dementia in tauopathies (Wilcock and Esiri, 1982; Arriagada et al., 1992; Mukaetova-Ladinska et al., 2000; Maruyama et al., 2013; Brier et al., 2016; Gomperts et al., 2016; Smith et al., 2016) and oligomeric tau species are implicated in the spread of tau aggregates through the brain (Clavaguera et al., 2009; Sanders et al., 2014). Tau is encoded by the *MAPT* gene, which produces six isoforms in the human nervous system through alternative splicing (Goedert et al., 1989). These isoforms range from 352 to 441 amino acid residues and contain either three (3R) or four (4R) tandem imperfect repeats, which are 31 of 32 residues in length (Crowther et al., 1989). Monomeric tau is able to stabilize microtubules and is also thought to play roles in signal transduction, actin interaction, and the binding of pericentromeric chromatin in the nucleus (Buée et al., 2000; Bukar Maina et al., 2016; Mansuroglu et al., 2016).

Early studies on tau filaments showed that they form canonical cross-β (amyloid) structures with an in-register parallel-β core (Berriman et al., 2003; Margittai and Langen, 2004). In recent years, structures of the cross-β cores of tau filaments extracted from patient tissue have been resolved thanks to advances in cryo-electron microscopy (cryo-EM). These structures showed that specific tauopathies such as corticobasal degeneration (CBD) have unique tau fibril cores, whereas filaments extracted from other diseases showed fibril core structures very similar to those found in AD (Fitzpatrick et al., 2017; Hallinan et al., 2021; Shi et al., 2021). This phenomenon of disease-specific fibril strains has been observed for other cross-β fibrils in disease including those formed by α-synuclein (Bousset et al., 2013; Mehra et al., 2021; Sawaya et al., 2021).

Despite advances in our understanding of patient-derived tau filaments, there is a need for *in vitro* models of these filaments to facilitate the screening of small molecules as potential biomarkers or therapeutics and allow the production of strain-specific antibodies.

Full-length tau is highly soluble and not very aggregation prone. Therefore, heparin or other polyanionic molecules have been used to induce aggregation *in vitro* (Goedert et al., 1996; Kampers et al., 1999; Pérez et al., 2001). However, a truncated tau (297-391), first recognized as the protease-resistant core of PHFs in AD (Jakes et al., 1991; Novak et al., 1993) was found to form filaments *in vitro* in the absence of any additives (Al-Hilaly et al., 2017). This fragment of tau is also termed dGAE, where “d” corresponds to the amino acid Ile-297 and “GAE” to the three C-terminal amino acids Gly-389, Ala-390, Glu-391 from the 441-residue tau isoform containing 2 N-terminal inserts and 4 tandem repeats (2N4R). Furthermore, tau(297-391) overlaps with the core of filaments resolved from *ex vivo* filaments by cryo-EM (Fitzpatrick et al., 2017), was shown to form PHF that resemble those observed in AD patients (Al-Hilaly et al., 2020), and later confirmed by cryo-EM to form an architecture identical to those from AD brain under certain conditions (Lövestam et al., 2022). Tau(297-391) has been shown to recruit endogenous tau in a cell model (Harrington et al., 2015) and is able to self-propagate by binding full-length tau reproducing the protease-resistant core following treatment with proteases (Wischik et al., 1996). The tau(297-391) sequence contains a single cysteine residue at position 322 and the oxidation/reducing conditions of assembly have been shown to have a profound effect on the assembly and morphology of the resulting filaments due to the formation of a disulphide bound dimer under non-reducing conditions (Al-Hilaly et al., 2017).

Here we examine the consequences of aggregation in a reducing vs. a non-reducing environment on the structure and dynamics of the tau(297-391) filaments and compare the structure of such filaments to *ex vivo* filaments from AD patients resolved by cryo-EM. Our results show that the filaments formed from tau(297-391) are well ordered so long as C322 is reduced. The comparison of our resonance assignment of tau(297-391) filaments formed under reducing conditions with cryo-EM structures of tau generally confirms that these filaments share the cross-β core structure with AD type *ex vivo* filaments.

## Materials and methods

### Protein expression and purification

Uniformly ^13^C-^15^N labeled tau(297-391) was expressed in *E. coli* following a protocol described by Marley and co-workers (Marley et al., 2001). The purification was conducted as previously described (Al-Hilaly et al., 2017, 2020). In short, after cell lysis and removal of the insoluble fraction by centrifugation, tau(297-391) was purified by heat treatment and cellulose phosphate ion exchange chromatography in MES buffer (pH 6.25). The clarified bacterial lysate was made to 0.75 M NaCl, 10 mM DTT and heated in a boiling water bath for 5 min. The precipitated proteins were removed by centrifugation and the supernatant fraction dialysed against the MES buffer (50 mM, pH 6.25). Protein fractions were eluted from cellulose phosphate using a KCl gradient from 0.1 to 1 M in a 50 mM MES (pH 6.25) buffer. The protein usually eluted between 0.3 and 0.5 M KCl and pooled fractions were dialyzed against 10 mM phosphate buffer (pH 6.8). To initiate filament formation, 300 μM of ^13^C-^15^N labeled tau(297-391) was incubated in phosphate buffer (10 mM, pH 7.4) with and without 1,4 dithiothreitol (DTT) (10 mM) and agitated at 400 oscillations per minute (Eppendorf Thermomixer C, Eppendorf, Germany) at 37°C for 48 h.

### Electron microscopy

Electron microscopy grids were prepared by placing 4 μl of the sample onto formvar/carbon-coated 400-mesh copper grids (Agar Scientific Ltd, UK), followed by two washes with 0.22-μm-filtered milli-Q water. Then, 4 μl of uranyl acetate (2% w/v) was added to the grid and left for 30 s before blotting. EM projection images were collected using a JEOL JEM1400-Plus Electron Microscope operated at 80 kV equipped with a Gatan OneView camera (4k × 4k). Images were recorded at 25 fps with drift correction using GMS3.

### NMR experiments and data analysis

Uniformly ^13^C-^15^N-labeled tau(297-391) filaments were centrifuged into 1.6 mm rotors using a home built packing tool similar to the one described by Böckmann et al. (2009). The following experimental details apply to all NMR experiments: an Agilent DD2 spectrometer with a proton frequency of 600 MHz equipped with a 1.6 mm triple-resonance MAS probe was used. The set-temperature for all experiments was 0°C. ^1^H, ^13^C, ^15^N hard pulses were done with rf-field strengths of 200, 100, and 50 kHz, respectively. High-power ^1^H decoupling was done using the XiX decoupling scheme (Detken et al., 2002) during direct and indirect time domains and CW decoupling was applied during DREAM and double CP recoupling steps all with a ^1^H rf-field strength of 140 kHz.

1D cross polarization (CP) and refocused INEPT experiments were recorded at 25 kHz MAS, a spectral width of 50 kHz and 1,024 acquisitions were added for each of these spectra. The initial ^1^H-^13^C CP was done with a contact time of 1 ms and rf-field strengths of 85 and 60 kHz on ^1^H and ^13^C, respectively. The 2D PARIS experiment used the same CP to create the initial ^13^C magnetization followed by a 500 ms of PARIS ^13^C mixing using a 10 kHz recoupling field on ^1^H and the *N* = 0.5 condition (Weingarth et al., 2009). Spectral widths were 50 kHz in both dimensions and 112 acquisitions were co-added for each of the 350 complex, indirect increments. The 2D DREAM was recorded at 35 kHz MAS with 4.5 ms of DREAM recoupling and 32 acquisitions were co-added for each of the 800 real, TPPI increments. The 2D NCA spectrum was recorded at 35 kHz MAS using a standard double CP (DCP) pulse sequence using an initial 1.5 ms ^1^H-^15^N CP with rf-field strengths of 85 kHz on ^1^H and 50 kHz on ^15^N followed by 8 ms of SPECIFIC CP (Baldus et al., 1998) with rf-field strengths of 40 and 5 kHz on ^15^N and ^13^C, respectively, and the ^13^C transmitter set to 52 ppm. Spectral widths were 50 kHz for ^13^C and 3 kHz for ^15^N and 128 acquisitions were co-added for each of the 40 complex, indirect increments. The NCO spectrum was acquired with the same rf-field strengths but the ^13^C transmitter set to 174 ppm instead and a mixing time of 9 ms. In addition, homonuclear decoupling using the LOW BASHD scheme (Struppe et al., 2013) was applied during t_1_. Spectral widths were 2.5 kHz for ^13^C and 3 kHz for ^15^N and 96 acquisitions were co-added for each of the 40 complex, indirect increments.

The NCOCA was recorded at 35 kHz MAS using a double CP followed by a DREAM sequence for homonuclear ^13^C-^13^C transfer (Detken et al., 2001). The ^1^H-^15^N and ^15^N-^13^C were the same as for the NCO 2D followed by 2.5 ms of DREAM recoupling at the HORROR condition of 17.5 kHz during which the ^13^C transmitter was moved to 115 ppm. Spectral widths were 50 kHz for the direct and 3 kHz both of the indirect dimensions, each of which was sampled with 32 indirect, complex points and 64 acquisitions per increments that were recorded using non-uniformly sampling with 25% coverage. The NCACO was recorded with the same pulse sequence as the NCOCA spectrum but using the double CP conditions used for the 2D NCA spectrum and the same DREAM recoupling conditions. Spectral widths were 50 and 5 kHz for the direct and indirect ^13^C dimensions, and 3 kHz for the ^15^N dimension. Thirty two acquisitions were co-added for each of the 32 indirect ^13^C and 20 indirect ^15^N increments that were acquired using non-uniformly sampling at 25% coverage.

The NCACB 3D was recorded at 25 kHz MAS using the same pulse sequence as the NCOCA spectrum. ^1^H and ^15^N rf-field strengths of 65 and 40 kHz were used for the 1st CP with a contact time of 1.5 ms and ^15^N and ^13^C rf-field strengths of 32.5 and 7.5 kHz for the 2nd CP with a contact time of 9 ms and the ^13^C transmitter set to 45 ppm. DREAM recoupling at the HORROR condition of 12.5 kHz was done for 3.5 ms for the final transfer step. Spectral widths were 50 and 6 kHz for the direct and indirect ^13^C dimensions, and 3 kHz for the ^15^N dimension and 56 acquisitions were co-added for each of the 50 indirect ^13^C and 26 indirect ^15^N increments that were acquired using non-uniformly sampling at 25% coverage.

The 3D CANcoCA experiment (see Supplementary Figure 1) was recorded at 35 kHz MAS using three consecutive CPs and a ^13^C-^13^C DREAM transfer step. The initial ^1^H-^13^C CP was done for 1ms using rf-field strengths of 85 and 50 kHz on ^1^H and ^13^C, respectively. The second and third CP were done for 6 ms with rf-field strength of 40 and 5 kHz on ^15^N and ^13^C, respectively. The CA-N transfer was done with the transmitter at 51 ppm and the NCO transfer with the transmitter at 167 ppm. The final DREAM transfer was done for 2.5 ms at the HORROR condition of 17.5 kHz with the transmitter set to 111 ppm.

All NMR time domain datasets were processed using the nmrPipe software package including NUS datasets, which were processed using the IST algorithm implemented in nmrPipe (Delaglio et al., 1995). Spectra were analyzed using the program CARA (Keller, 2004) and plotted using the nmrglue python package (Helmus and Jaroniec, 2013).

## Results

### Tau(297-391) filaments formed under reducing conditions are more ordered and less dynamic

**FIGURE 1:**
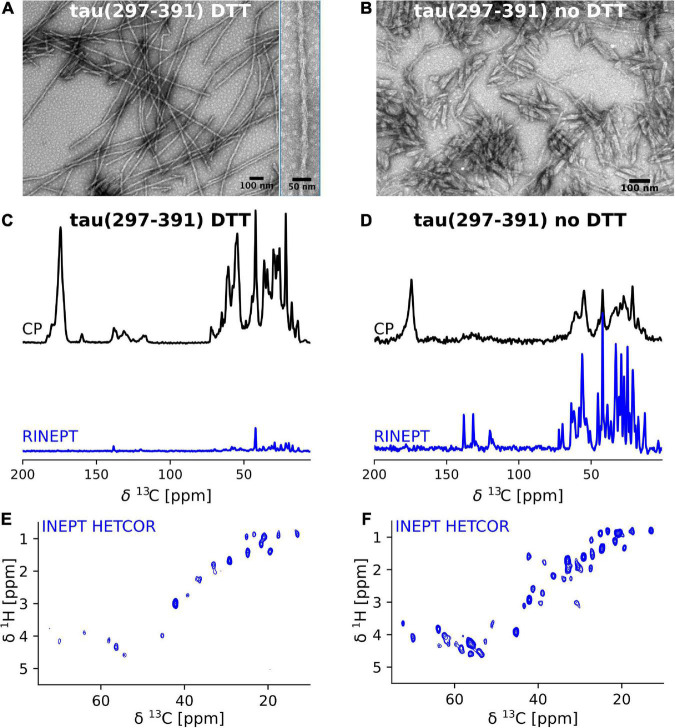
Tau(297-391) filaments formed in the presence of DTT are much more rigid and ordered compared to filaments formed without DTT. **(A,B)** EM images of solid-state NMR samples of tau(297-391) filaments formed with and without DTT. **(C–F)** Solid-state NMR spectra of tau(297-391) filaments formed in the presence **(C,E)** or absence of DTT **(D,F)**. **(C,D)** 1D ^13^C spectra recorded using ^1^H-^13^C cross polarization (CP, black) and a refocused INEPT transfer (RINEPT, blue) are shown. The CP experiment shows the more static domains of the sample whereas the RINEPT experiment only shows highly dynamic parts of the sample under the conditions used. The strong intensity of the CP spectrum and comparably negligible intensity of the RINEPT spectrum of the filaments made with DTT suggests that the majority of the sample is part of the rigid fibril core. In contrast, the intense RINEPT spectrum of the filaments formed in the absence of DTT suggest that this sample contains a lot of dynamic disorder. **(E,F)** The 2D ^1^H-^13^C INEPT-HETCOR spectra sensitive to dynamic domains confirm this tendency showing many more signals for the sample made in the absence of DTT.

We previously showed that tau(297-391) formed filaments with different morphologies dependent on the presence of DTT as a reducing agent (Al-Hilaly et al., 2017). To follow up on this finding, we asked how assembling the protein under reducing conditions affects the structure and dynamics of the monomeric constituents of tau(297-391) filaments. To answer this question, we prepared uniformly ^13^C-^15^N labeled tau(297-391) filaments with and without the addition of DTT for solid-state NMR analysis (see Section “Materials and methods”). The EM images of our NMR samples in Figures 1A,B show that filaments made from labeled tau(297-391) in the presence of DTT closely resemble those found in AD. They are twisted with a repeat distance of 71.3 ± 5 nm. Also, they are generally longer than the fibrils made in the absence of DTT confirming previous results (Al-Hilaly et al., 2017, 2020). Figures 1C,D show 1D ^13^C solid-state NMR spectra of both samples. We used two different solid-state NMR experiments to highlight regions of different dynamics. The cross polarization (CP) experiment is sensitive to relatively static portions of the sample, which in the case of amyloid fibrils is usually the cross-β core and static framing sequences. The refocused INEPT (RINEPT) experiment, on the other hand, only shows signals coming from regions of considerable dynamics (Heise et al., 2005; Siemer et al., 2006a; Matlahov and van der Wel, 2018; Siemer, 2020). Based on these 1D spectra, the differences between the two fibril types were striking: The filaments formed in the presence of DTT give strong and relatively narrow signals in the CP and only few weak signals in the RINEPT spectrum, indicating that the majority of the tau(297-391) residues are part of a well ordered, static fibril core. In contrast, the filaments formed without DTT give a CP spectrum that is relatively weaker and broader and an RINEPT spectrum that shows many, intense resonances, indicating that these filaments have a less ordered fibril core and that large parts of the sample are in a much more dynamic state.

These findings are further supported by the 2D INEPT HETCOR spectra shown in Figures 1E,F. These spectra, which are 2D versions of the 1D RINEPT spectrum, show many more and more intense signals for the tau(297-391) filament sample formed without DTT compared to the sample formed in the presence of DTT.

### Resonance assignment of tau(297-391) filaments formed under reducing conditions

Further atomic-resolution NMR analysis of these filaments requires resonance assignments. Because the CP signals of non-DTT tau(297-391) filaments were weak and relatively broad, and CP-based 2D spectra showed only a few weak cross peaks, their assignment was not feasible. Therefore, we focused on tau(297-391) filaments formed in the presence of DTT. To assign the resonances in the static fibril core, we recorded a standard set of ^13^C based solid-state NMR 2D and 3D experiments, namely, 2D DREAM, NCO, and NCA and 3D NCOCA, NCACB, NCACO, and CANcoCA spectra (Siemer et al., 2006b; Schuetz et al., 2010). We also recorded a ^13^C-^13^C 2D PARIS (Weingarth et al., 2009) spectrum with 500 ms mixing time to confirm some of our assignments.

**FIGURE 2:**
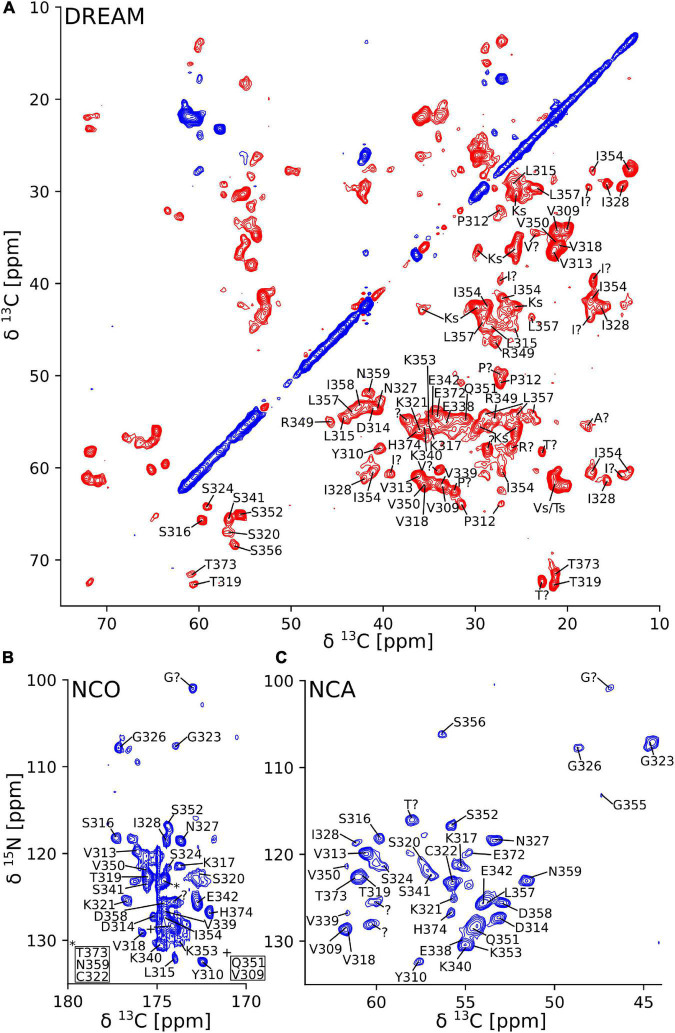
Two-dimensional solid-state NMR spectra of tau(297-391) filaments made with DTT show narrow lines and one set of cross peaks indicative of a homogenous and well-ordered structure. **(A)** 2D ^13^C-^13^C DREAM spectrum recorded at 35 kHz MAS. Negative and positive cross peaks are shown in red and blue, respectively. **(B)** 2D ^15^N-^13^C NCO spectrum recorded at 35 kHz MAS using LOW BASHD decoupling during t_2_. **(C)** 2D NCA spectrum recorded under conditions similar to the NCO spectrum. Residue-specific resonance assignments are indicated showing an almost complete assignment of these spectra.

**FIGURE 3:**
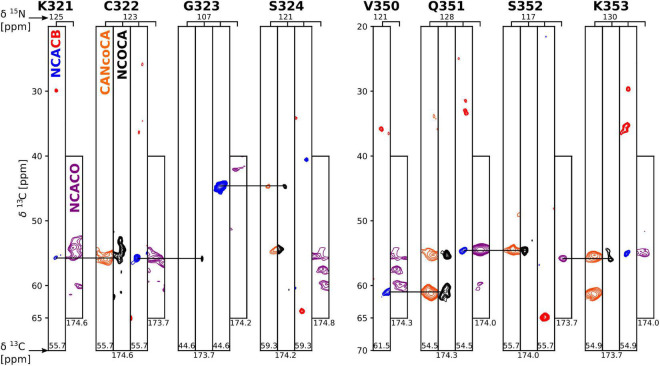
Strip plot of 3D spectra used for resonance assignment. Strips through 3D CANcoCA (orange), NCOCA (black), NCACB (blue = positive; red = negative), and NCACO (purple) spectra. The ^15^N shift and either CA or CO shift of each strip are indicated. Horizontal lines indicate the connectivities *via* common CA/CA-1 resonances.

The 2D DREAM, NCA, and NCO spectra of the tau(297-391)+DTT filaments illustrate the good spectral resolution of this sample (Figure 2). Slices through the 2D DREAM are shown in Supplementary Figure 2. A comparable 2D DREAM of non-DTT tau(297-391) filaments having fewer and broader cross peaks is shown in Supplementary Figure 3. As indicated by the residue-specific labels in Figure 2, we were able to assign the large majority of the cross peaks in these 2D spectra, with the help of the 3D spectra, which are illustrated in Figure 3. We identified the following residues in our assignment starting with V309 and Y310 followed by P311-S324, G326-I328, E338-E342, R349-N359, and E372-H374. In addition, we identified a PGGG fragment that is either P332-G335, or P364-G367, or both. The assignment includes backbone ^13^C and ^15^N resonances as well as side chain ^13^C resonances for most of the assigned residues as can be seen from the DREAM spectrum in Figure 2A. The residues of tau(297-391) that were not part of our assignment, could either not be assigned with confidence in our analysis (i.e., the few residues labeled with “?” in Figure 2), didn’t give intense enough signals in our spectra because they were too dynamic or in regions of high static disorder, or were hidden by overlap with other residues. Because of the narrow linewidth and the low intensity of our RINEPT spectra (Figure 1), signal overlap likely contributes to most of the missing assignments. For example there are three PGGG sequences in tau(297-391), which could not be assigned uniquely. In addition, only 4 of the 14 lysines in tau(297-391) could be assigned most likely because of signal overlap.

### Comparison with tau fibril core structures

Because chemical shifts, in particular of Cα and Cβ atoms, are very sensitive to secondary structure (Spera and Bax, 1991), we were now able to ask: How does our solid-state NMR assignment compare to the structure of tau fibril strains isolated from different tauopathies? To address this question, we chose two different approaches: firstly we derived chemical shifts from existing tau fibril core structures and compared them to our measured chemical shifts, and secondly we derived dihedral angles from our NMR assignment and compared them to the dihedral angles of the same tau core structures.

For the first approach, we used the program ShiftX2 (Han et al., 2011), which derives chemical shifts from a given PDB file. We focused on Cα and Cβ chemical shifts (δ) known to be most sensitive to secondary structure (Spera and Bax, 1991) and examined all tau fibril structures deposited to the PDB, whose cores included the residues found in our solid-state NMR assignment. We then compared the thus calculated chemical shifts (*Cal*) to our NMR assignment (*NMR*) *via* the sum of the absolute per residue Cα and Cβ chemical shift difference for every assigned residue *i*


(1) $$\Sigma_{C\,S}=\sum_{i}\left|\delta \,C\,\alpha_{i}^{C\,a\,l}-\delta \,C\,\alpha_{i}^{N\,M\,R}\right|+\left|\delta \,C\,\beta_{i}^{C\,a\,l}-\delta \,C\,\beta_{i}^{N\,M\,R}\right|.$$

**FIGURE 4:**
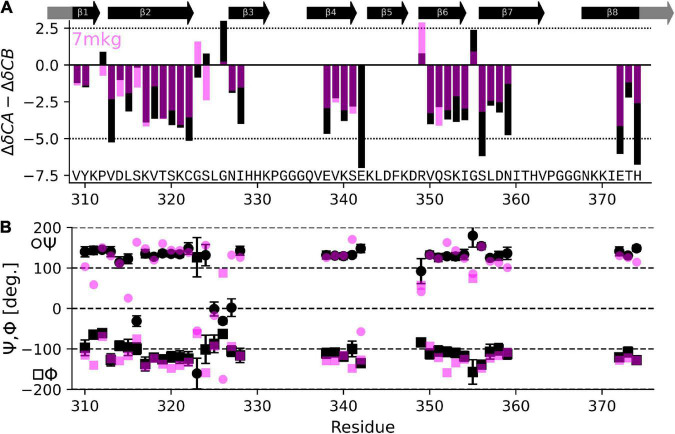
Comparison of NMR assignment and existing cryo-EM structures. **(A)** Difference in CA and CB secondary chemical shift (*S* = ΔδCα−ΔδCβ) as derived from the NMR assignment (black) and calculated from the tau PrP-CAA SF cryo-EM structure (PDB entry 7mkg, fuchsia) using the program ShiftX2. Both secondary shifts overlap well indicating a similar structure. However, there are notable exceptions, specifically P312, G324, S324, G326, and E342. **(B)** Psi (Ψ), phy (Φ) dihedral angles calculated from the NMR assignment using the program TALOS-N (black) and taken from the cryo-EM structure 7mkg.

The result in Table 1 show that the PDB entry 7mkg i.e., the core of a fibril extracted from a patient with PrP cerebral amyloid angiopathy (Hallinan et al., 2021) resulted in the lowest chemical shift difference (for the complete set of results please see Supplementary Table 1). To visualize the per-residue similarity in chemical shift, we calculated the difference between the Cα and Cβ secondary chemical shifts (*S* = ΔδCα − ΔδCβ) for both the chemical shifts derived from the 7mkg structure and our assignment. As can be seen from Figure 4A, there is, with few exceptions, a good agreement in sign and amplitude of the individual *S* values. The sign of *S* is indicative of secondary structure where positive values are found in helical structures and negative values in extended conformations such as β-strands. However, all tau core structures are dominated by β-sheets resulting in negative *S* values for most residues. What really distinguishes these cores from a secondary structure perspective are the locations of the kinks i.e., the regions that deviate from β-strand conformation. With this in mind, we developed another metric Σ_*A*_ to compare existing tau core structures with our NMR assignment by calculating the number of residues for which the sign of *S* was different between the two.


(2) $$\Sigma_{A}=\sum_{i}A_{i}\text{with}A_{i}:=\{1\text{if}\left(S_{i}^{C\,a\,l}\cdot S_{i}^{N\,M\,R}\right)<0,$$

$$\;\;0\mathrm{if}\left(S_{i}^{C\,a\,l}\cdot S_{i}^{N\,M\,R}\right)\geq 0\}$$

**TABLE 1:** Comparison of NMR chemical shift assignment with existing tau fibril structures indicates that tau(297-391) fibrils adopt the AD fold.

PDB	Σ_CS_ (ppm)	Σ_A_	Σ_ΨΦ_ (deg)	Fold
7p6d	65.8414	4	3,648	AGD
7qjv	73.6912	4	3,361	AD
7mkg	62.9429	5	2,848	AD
5o3o	74.1779	5	3,146	AD
7mkf	71.8944	6	2,883	AD
7nrs	74.7738	6	2,986	AD
7nrt	74.9086	6	2,987	AD
7nrx	83.0367	6	2,972	AD
5o3t	88.8657	6	3,827	AD
7p6e	97.3397	6	3,786	AGD
7p6c	69.0672	7	2,661	LNT

Subset of results from the three metrics introduced in the text, namely, Σ_CS_ (equation 1), Σ_A_ (equation 2), and Σ_ΨΦ_ (equation 3). Best (i.e., minimal) values for each metric are underlined. The table is sorted according to Σ_A_ and the eleven best results are shown together with the corresponding PDB identification codes and the type of fold (AGD, argyrophilic grain disease; AD, Alzheimer’s disease; LNT, limbic-predominant neuronal tauopathy).

The best results of this calculation are shown in Table 1. In this case two PDB entries give the best (i.e., lowest) score: 7qjv a structure of recombinant tau(297-391) (Lövestam et al., 2022) and 7p6d the structure of tau fibrils from an argyrophilic grain disease patient (Shi et al., 2021) (a complete set of results can be found in Supplementary Table 1).

For the second approach to compare known tau fibril structures with our NMR assignment, we calculated dihedral Φ,Ψ angles from our assignment (*NMR*) using the program TALOS-N (Shen and Bax, 2013). We then derived Ψ,Φ angles from tau core PDBs (*Cal*) using the program PyRosetta (Chaudhury et al., 2010). Finally, we calculated the sum of the absolute per residue difference in dihedral angles, namely


(3) $$\Sigma_{\Psi \,\Phi }=\sum_{i}\left|\Phi_{i}^{C\,a\,l}-\Phi_{i}^{N\,M\,R}\right|+\left|\Psi_{i}^{C\,a\,l}-\Psi_{i}^{N\,M\,R}\right|.$$

Table 1 also shows results of this calculation (the complete set is shown in Supplementary Table 1). In this case PDB entry 7p6c, a structure extracted from limbic-predominant neuronal inclusion body tauopathy (Shi et al., 2021), gave the lowest overall difference. Figure 4B illustrates the Ψ,Φ angles calculated from the NMR chemical shifts (black) and 7mkg (fuchsia).

**FIGURE 5:**
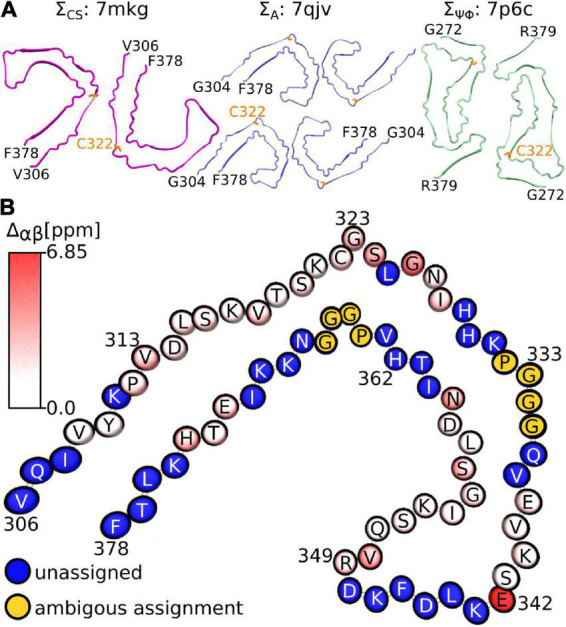
Fibril structures best fitting to NMR assignment. **(A)** Top view of fibril structures that fit best using the Σ_CS_ metric (7mkg), the Σ_A_ metric (7qjv, 7p6d not shown), and the Σ_ΨΦ_ metic (7p6c). The N and C-terminal residues are indicated in black, C322 is highlighted in orange. **(B)** Birds-eye view on chain A of PDB entry 7mkg. Assigned residues are colored according to the per residue Cα and Cβ chemical shift difference calculated using the program ShiftX2 i.e., $\Delta_{\alpha \,\beta }=|\delta C\alpha_{i}^{C\,a\,l}-\delta C\alpha_{i}^{N\,M\,R}|+|\delta C\beta_{i}^{C\,a\,l}$$-\delta C\beta_{i}^{N\,M\,R}|$. Unassigned residues are shown in blue. The PGGG sequences that were ambiguously assigned are shown in yellow. Residue number and type are indicated. Panel **(B)** was made with the help of the program VMD.

Considering that the AD fold cryo-EM structure of recombinant tau(297-391), formed under very similar conditions to ours, should be the “correct” answer, it is surprising that our three metrics result in good scores for non-AD tau core structures (see Figure 5A). Although none of our metrics are perfect, Σ_*A*_ seems to produce the most reliable results with 8 of the 10 top scoring structures having AD folds in contrast to only 2 of the 10 bottom scoring structures. When looking at all three metrics, PDB entry 7mkg has the overall best fit with the lowest Σ_CS_, second lowest *S*, and 4th lowest Σ_ΨΦ_.

Figure 5B illustrates the location of the residues in our assignment on the AD fold of tau (7mkg) and indicates the per residue difference between the measured (NMR) and calculated Cα and Cβ chemical shifts i.e.


(4) $$\Delta_{\alpha \,\beta }=\left|\delta \,C\,\alpha_{i}^{C\,a\,l}-\delta \,C\,\alpha_{i}^{N\,M\,R}\right|+\left|\delta \,C\,\beta_{i}^{C\,a\,l}-\delta \,C\,\beta_{i}^{N\,M\,R}\right|.$$

Overall, the agreement is very good as seen in Figure 4A, with the biggest difference found for E342 followed by G326 and S324.

## Discussion

Cysteine residues in in-register parallel β-sheet structures result in a continuous array of thiols whose oxidation state is likely to influence both the formation and structure of the fibril. We previously showed that the oxidation state of C322 has important consequences for tau(297-391) filament forming kinetics (Al-Hilaly et al., 2017). Electron micrographs of these two filament types had shown a similar appearance with the non-DTT filaments being much shorter (Figure 1 and Al-Hilaly et al., 2017). Here, we followed up on these investigations using solid-state NMR on tau(297-391) filaments formed both in the presence and absence of DTT as a reducing agent. We found these structures to be remarkably different. For tau(297-391)+DTT, static well-ordered filaments were formed with few dynamic residues indicating that a large fraction of the tau(297-391) monomer is part of the fibril core. In contrast, tau(297-391) filaments formed in the absence of DTT were much less ordered as indicated by the relatively broader CP spectrum of these filaments. In addition, the relatively more intense RINEPT spectra indicated that a much larger portion of the sample was dynamic. This could either be the result of a smaller fibril core and a larger fraction of tau(297-391) remaining intrinsically disordered in the filament state, or a larger fraction of tau(297-391) staying as dimers in solution in equilibrium with the filament. These results support our previous hypothesis that disulfide-based tau dimerization is diametral to fibril formation which is in line with findings indicating that disulfide formation results in off-pathway aggregates (Walker et al., 2012). However, there are several studies arguing that disulfide formation is an important step in tau fibril formation (Schweers et al., 1995; Barghorn and Mandelkow, 2002; Furukawa et al., 2011; Kim et al., 2015) and that the inhibitory effect of methylthioninium chloride is the prevention of the formation of on-pathway disulfide bonds (Akoury et al., 2013; Crowe et al., 2013). In contrast to our present work, these somewhat contradictory results were obtained on full-length tau, which has multiple cysteine residues, aggregated in the presence of heparin. In addition, Lövestam and co-workers showed that a 50:50 mixture of tau(266-391) and tau(297-391) could form fibrils with disulfide bonds even in the presence of DTT but the resulting structures were different from the AD fold. The tau(297-391) fibrils in the same study did not show any disulfide bonds (Lövestam et al., 2022).

The use of heparin to form *in vitro* PHF also complicates the comparison of our solid-state NMR assignment with other assignments of tau and tau fragments. There are two solid-state NMR assignments of filaments formed by the tau fragment K19 in the presence of heparin (Andronesi et al., 2008; Daebel et al., 2012). Daebel and co-workers were able to assign residues V306-S324 and observed a doubling of the resonances close to C322 caused by the different oxidation state of this cysteine. A C322A mutant removed this splitting and improved the overall quality of the spectra (Daebel et al., 2012). Dregni and co-workers presented the assignment of 0N4R and 0N3R filaments formed in the presence of heparin (Dregni et al., 2019, 2021). They identified the static core of 0N4R filaments to roughly be where *in vivo* derived fibril cores were located i.e., from residue 262 to 361 similar to our assignment which goes from 309 to 374. However, the fibril core of 0N3R extended far beyond current tau core structures spanning from R1 until the end of the C-terminus (261–441). Although all solid-state NMR assignments of filaments formed *in vitro* generally agree in the overall β-sheet structure of the core, they differ significantly in extent of their assignment. Even in the regions of tau that were assigned, the assignments differ in their per-residue coverage. Interestingly, none of these assignments was able to identify any of the PGGG fragments, probably because of their increased flexibility and repetitive nature. Supplementary Figure 4 shows the chemical shift comparison between our assignment of tau(297-391) and the assignment of 0N3R filaments made in the presence of heparin (BMRB ID 50785). Aside from some outliers, the ^13^C chemical shifts generally agree with each other. There are more pronounced differences for the ^15^N chemical shifts. Unfortunately, none of the other tau filament assignments were deposited in the BMRB.

The chemical shift and dihedral angle based comparison of our NMR assignment with cryo-EM structures of mostly patient derived tau fibril cores was not able to establish a unique link between our assignment and one known structure. This is surprising because we expected the cryo-EM structure of recombinant tau(297-391), formed under almost identical conditions to ours, to be the best match (Lövestam et al., 2022). However, it is important to note that our comparison is limited by the precision of the chemical shift prediction with the program Shiftx2 [with an RMSD for ^13^C shifts in the order of 0.5 ppm (Han et al., 2011)], the dihedral angle prediction with the program TalosN [with a Φ,Ψ RMSD of about 12° (Shen and Bax, 2013)], and, of course, the quality of the respective structure and of our assignment. Nonetheless, our analysis provides support for tau(297-391) filaments made in the presence of DTT adopting the AD fold. Our data suggests that comparing the position of the kinks in the β-strands by calculating the number of residues for which *S* has a different sign might be the best metric to compare NMR assignments with existing fibrils structures. Ultimately, this analysis highlights that the difference in fibril strains lies in large parts in changes to tertiary contacts of very similar secondary structure elements. In other words, most tau fibril strains are very similar in where their β-strand are located but different in how the resulting β-sheets fold back onto each other. With this in mind, our metrics would easily be able to distinguish structures with larger differences in secondary structure content. To better distinguish structures that are very similar in secondary structure content as tau fibril strains, the measurement of a few key tertiary contacts either using NMR or EPR would be of advantage.

A limitation of tau(297-391) as a tau fibril model is that it represents only the most static core region of the AD fold structure. The fibril formed from full-length isoforms includes other static portions besides the core as e.g., evidenced by additional densities in cryo-EM maps (Fitzpatrick et al., 2017) and regions with static, extended structure in the 0N3R NMR assignment (Dregni et al., 2021). In addition, full-length tau filaments include the intrinsically disordered framing sequences that are known as the “fuzzy coat” in the case of tau (Wischik et al., 1988; Wegmann et al., 2013). Nevertheless, the AD fibril core represented by tau(297-391) is the binding site of powerful inhibitors of tau aggregation such as epigallocatechin gallate (EGCG) and methylthioninium, which are also able to inhibit and disaggregate preformed tau filaments (Wischik et al., 1996; Seidler et al., 2022). Identifying the binding sites of potential drugs and biomarkers provides an important step toward finding new therapeutic and diagnostic tools in AD and other tauopathies. The NMR assignment presented here could form the basis of NMR-assisted screening and characterization of such drug candidates and biomarkers.

## Data availability statement

The datasets presented in this study can be found in online repositories. The names of the repository/repositories and accession number(s) can be found below: https://bmrb.io, BMRB ID 51483.

## Author contributions

YA-H optimized and prepared the samples and contributed to managing the project. CH packed the NMR sample and measured NMR data. JER expressed and purified ^13^C, ^15^N labeled tau(297-391). CRH, JMDS, and CMW contributed to the conception of the study and reviewed drafts of the manuscript. LCS managed the project, co-conceived the idea, and contributed to writing the manuscript. AS measured, processed, and analyzed NMR data and wrote the manuscript. All authors read and approved the final manuscript.
