# Supplementary material for: Solid-state NMR of paired helical filaments formed by the core tau fragment tau(297-391)
Source: Front Neurosci. 2022 Dec 8;16:988074. doi: 10.3389/fnins.2022.988074 (PMC9774000; doi:10.3389/fnins.2022.988074)
Supplement: Supplementary file 1 [file Data_Sheet_1.PDF]

Table S1: Comparison of NMR chemical shift assignment with existing tau fibril structures indicates that tau(297-391) fibrils adopt the AD fold. The following metrics were calculated for each of the structures identified by their PDB identification codes: Sum of the absolute per residue Cα and Cβ chemical shift (δ) differences between solid-state NMR assignment (*NMR*) and chemical shifts calculated using ShiftX2 [1] (*Cal*) over every assigned residue *i*

$$\Sigma_{CS} = \sum_i \left|\delta C\alpha_i^{Cal} - \delta C\alpha_i^{NMR}\right| + \left|\delta C\beta_i^{Cal} - \delta C\beta_i^{NMR}\right| . \quad (1)$$

Number of residues for which the difference in secondary chemical shift (Δδ) between Cα and Cβ (*S=ΔδCα-ΔδCβ*) has a different sign when calculated using ShiftX2 as compared to the NMR assignment.

$$\Sigma_A = \sum_i A_i \text{ with } A_i := \left\{1\ if\ (S_i^{Cal} \cdot S_i^{NMR}) < 0,\ 0\ if\ (S_i^{Cal} \cdot S_i^{NMR}) \geq 0\right\} \quad (2)$$

Sum of the absolute per residue Ψ, Φ differences as calculated from the solid-state NMR assignment using TALOS-N (*NMR*) and calculated from the PDB structure (*Cal*)

$$\Sigma_{\Psi\Phi} = \sum_i \left|\Phi_i^{Cal} - \Phi_i^{NMR}\right| + \left|\Psi_i^{Cal} - \Psi_i^{NMR}\right|. \quad (3)$$

Best (i.e. minimal) values for each metric are underlined and the table is sorted according to Σ_A. In addition, the table lists the fibril source and fold type where the abbreviations are defined as follows: AGD, argyrophilic grain disease; PrP-CAA, PrP cerebral amyloid angiopathy; AD, Alzheimer's disease; PART, primary age-related tauopathy; LNT, limbic-predominant neuronal tauopathy; CTE, chronic traumatic encephalopathy; GSS, gerstmann-sträussler-scheinker disease; GGT, globular glial tauopathy; CBD, corticobasal degeneration; PSP, supranuclear palsy.

PDB	Σ_{CS} [ppm]	Σ_A	$\Sigma_{\Psi\Phi}$ [deg]	tau construct	Disease / Source	Fold
7p6d	65.8414	<u>4</u>	3648	4R	AGD	AGD
7qjv	73.6912	<u>4</u>	3361	tau(297-391)	Recombinant	AD / Quadruple
7mkg	<u>62.9429</u>	5	2848	3R+4R	PrP-CAA	AD / Straight
5o3o	74.1779	5	3146	3R+4R	AD	AD / Paired
7mkf	71.8944	6	2883	3R+4R	PrP-CAA	AD / Paired
7nrs	74.7738	6	2986	3R+4R	PART	AD / Straight
7nrt	74.9086	6	2987	3R+4R	PART	AD / Straight
7nrx	83.0367	6	2972	3R+4R	AD	AD / Straight
5o3t	88.8657	6	3827	3R+4R	AD	AD / Straight

7p6e	97.3397	6	3786	4R	AGD	AGD
7p6c	69.0672	7	2661	4R	LNT	LNT
7p6b	73.1256	7	2723	4R	LNT	LNT
6nwp	75.0648	7	3314	4R	CTE	CTE
7nrq	76.0444	7	3093	3R+4R	PART	AD / Paired
7mkh	76.1057	7	2916	3R+4R	GSS	AD / Paired
7p67	83.657	7	3466	4R	GGT	GGT
6tjo	94.4223	7	3707	4R	CBD	CBD
7nrv	73.4251	8	2972	3R+4R	AD	AD / Paired
7p68	77.7252	8	3010	4R	GGT	GGT
5o3l	95.7989	8	3454	3R+4R	AD	AD /Paired
6tjx	96.9895	8	3474	4R	CBD	CBD
7p6a	76.5164	9	2754	4R	LNT	LNT
7p65	82.3178	9	3018	4R	PSP	PSP
7p66	89.1693	11	2963	4R	GGT	GGT
6nwq	90.8825	11	3314	4R	CTE	CTE

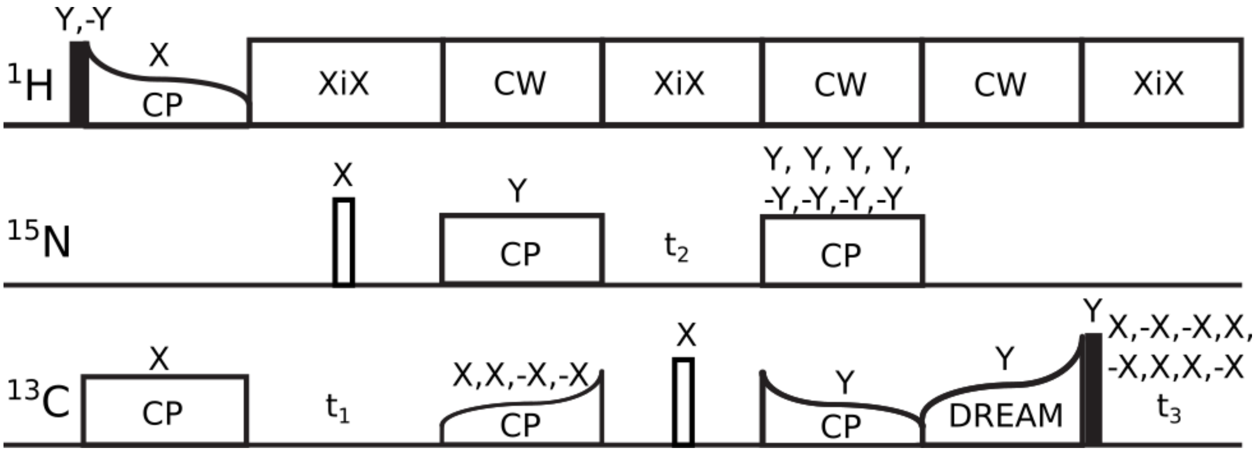

Figure S1: Pulse sequence for 3D NCAcoCA experiment. 90° and 180° pulses are shown as black and white rectangles, other pulse sequence elements are annotated and phases of individual pulses are given except for decoupling elements.

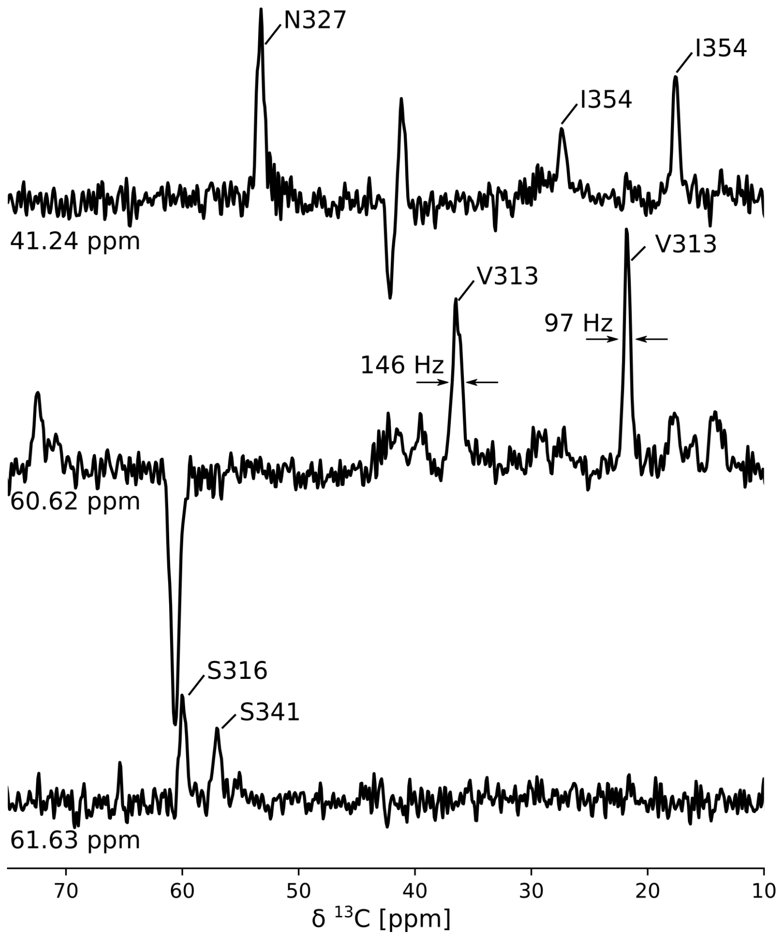

Figure S2: 1D slices through 2D DREAM spectrum illustrate quality of tau(297-391)+DTT sample. 1D slices through the 2D DREAM spectrum shown in Fig. 2A, which was processed without window function in the direct dimension for the present figure. PPM positions in the indirect frequency domain, assignments of cross peaks, and linewidths for V313 Cβ and Cγ resonances are indicated.

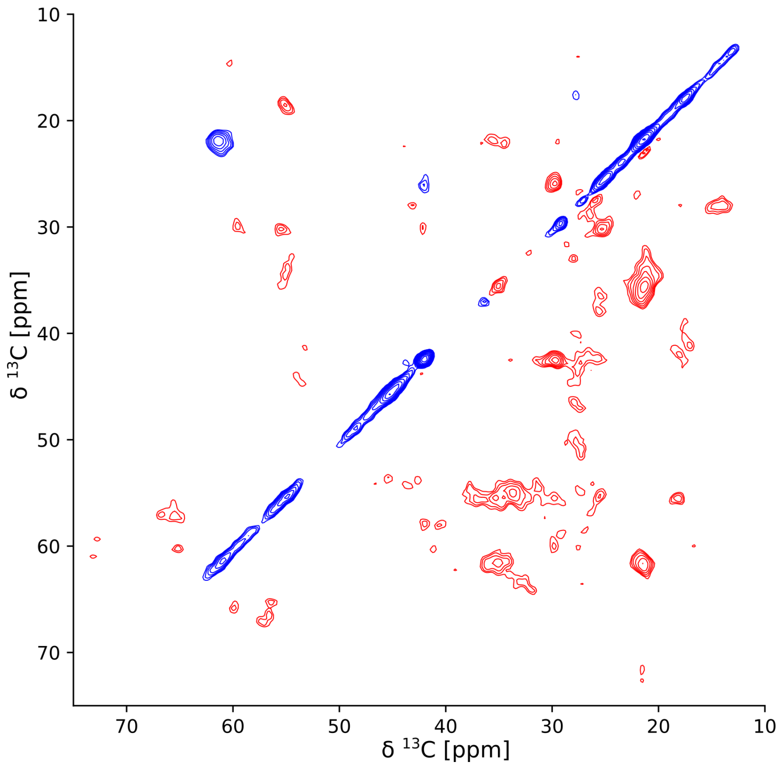

Figure S3: 2D DREAM spectrum of non-DTT tau(297-391) filaments shows fewer and broader cross peaks compared to the spectrum in Fig. 2A. 2D ^{13}C-^{13}C DREAM spectrum recorded at 35 kHz MAS, -10°C. Negative and positive cross peaks are shown in red and blue, respectively.

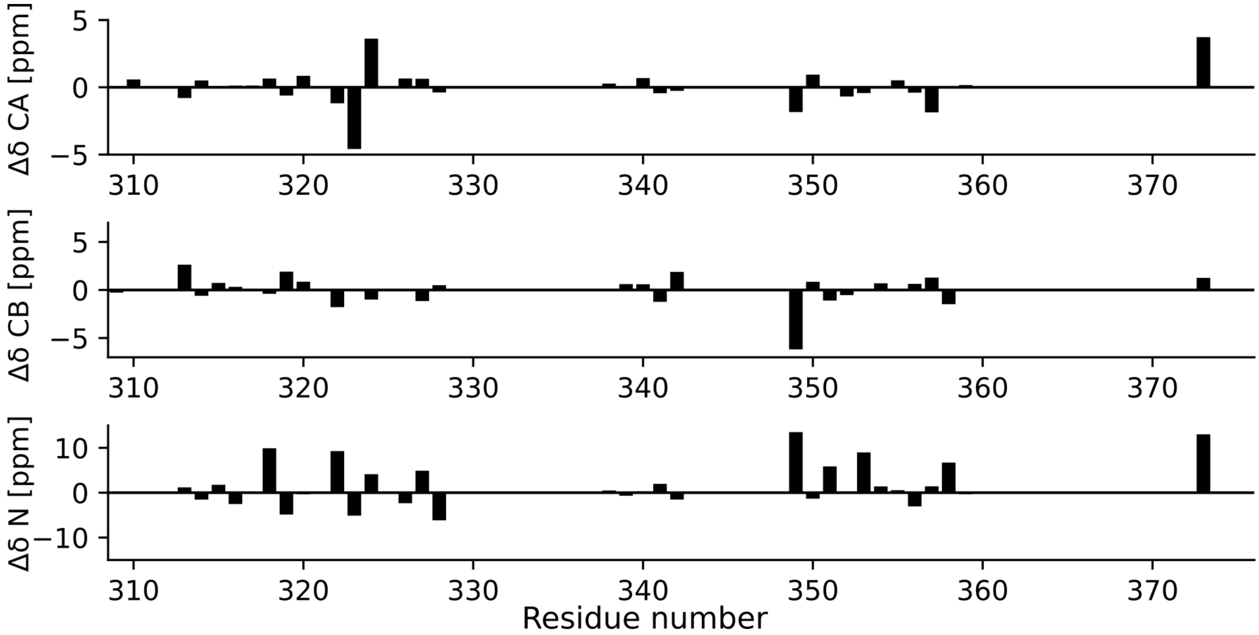

Figure S4: Comparison of 0N3R tau and tau(297-391) assignment. Bar graphs illustrating the difference in CA, CB, and N chemical shifts (Δδ) of our assignment of tau(297-391) fibrilized in the presence of DTT (BMRB ID 51483) and 0N3R tau fibrilized in the presence of heparin (BMRB ID 50785).
